# Supplementary material for: Characteristics of the AT-Hook Motif Containing Nuclear Localized (AHL) Genes in Carrot Provides Insight into Their Role in Plant Growth and Storage Root Development
Source: Genes (Basel). 2021 May 18;12(5):764. doi: 10.3390/genes12050764 (PMC8157401; doi:10.3390/genes12050764)
Supplement: Supplementary file 1 [file genes-12-00764-s001.zip › Figure_S1.pdf]

XP_017233497.1
Prediction: Signal peptide (Sec/SPI)

Cleavage site between pos. 29 and 30: ASG-LP. Probability: 0.4727

Protein type	Signal Peptide (Sec/SPI)	Other
Likelihood	0.56	0.44

Download: PNG / EPS / Tabular

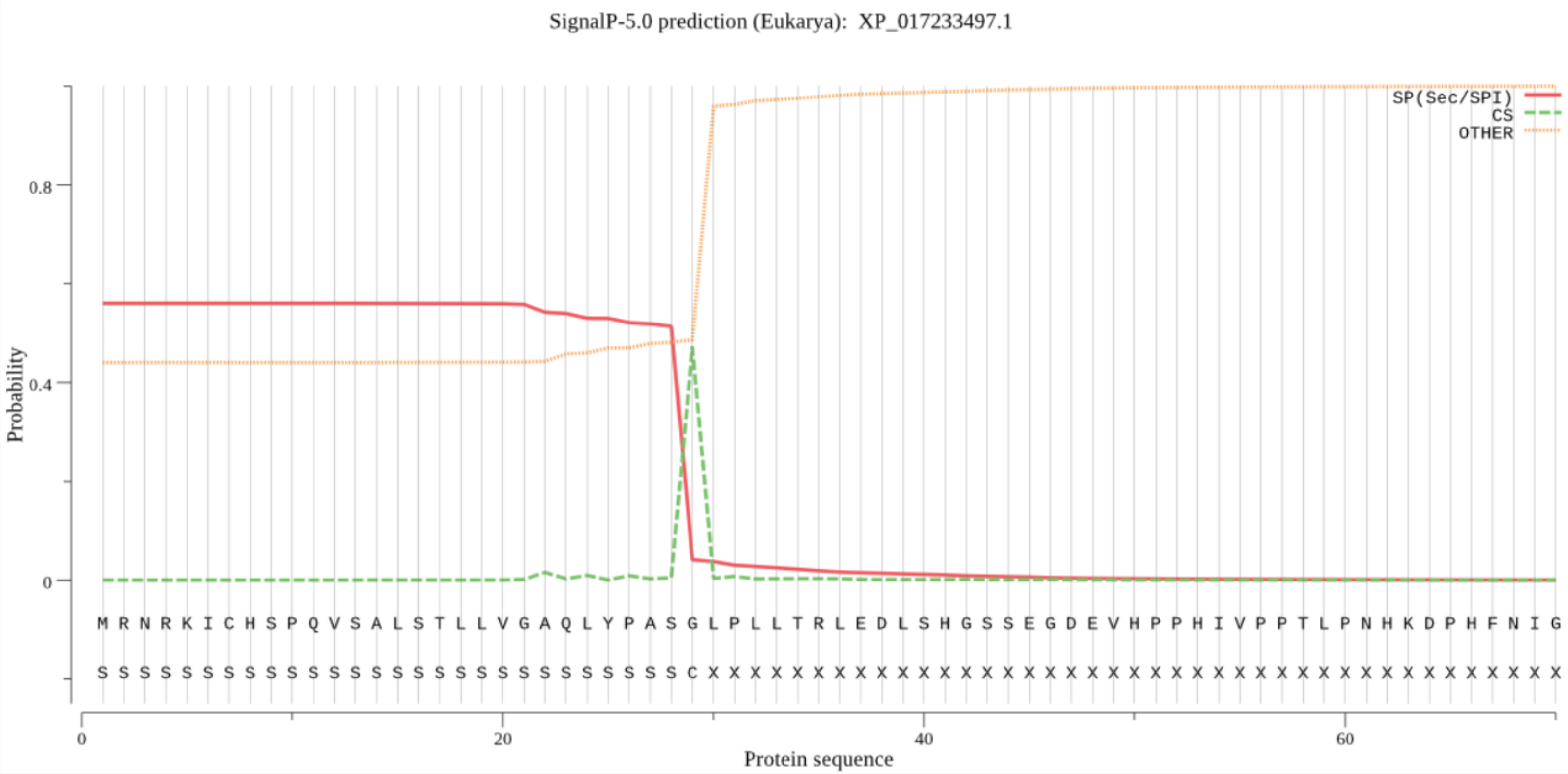

Figure S1. A graph showing the presence of a signal peptide in XP_017233497.1 indicating a possible extracellular localization of DcAHL9.
