# Supplementary material for: Characteristics of the AT-Hook Motif Containing Nuclear Localized (AHL) Genes in Carrot Provides Insight into Their Role in Plant Growth and Storage Root Development
Source: Genes (Basel). 2021 May 18;12(5):764. doi: 10.3390/genes12050764 (PMC8157401; doi:10.3390/genes12050764)
Supplement: Supplementary file 1 [file genes-12-00764-s001.zip › Figure_S2.pdf]

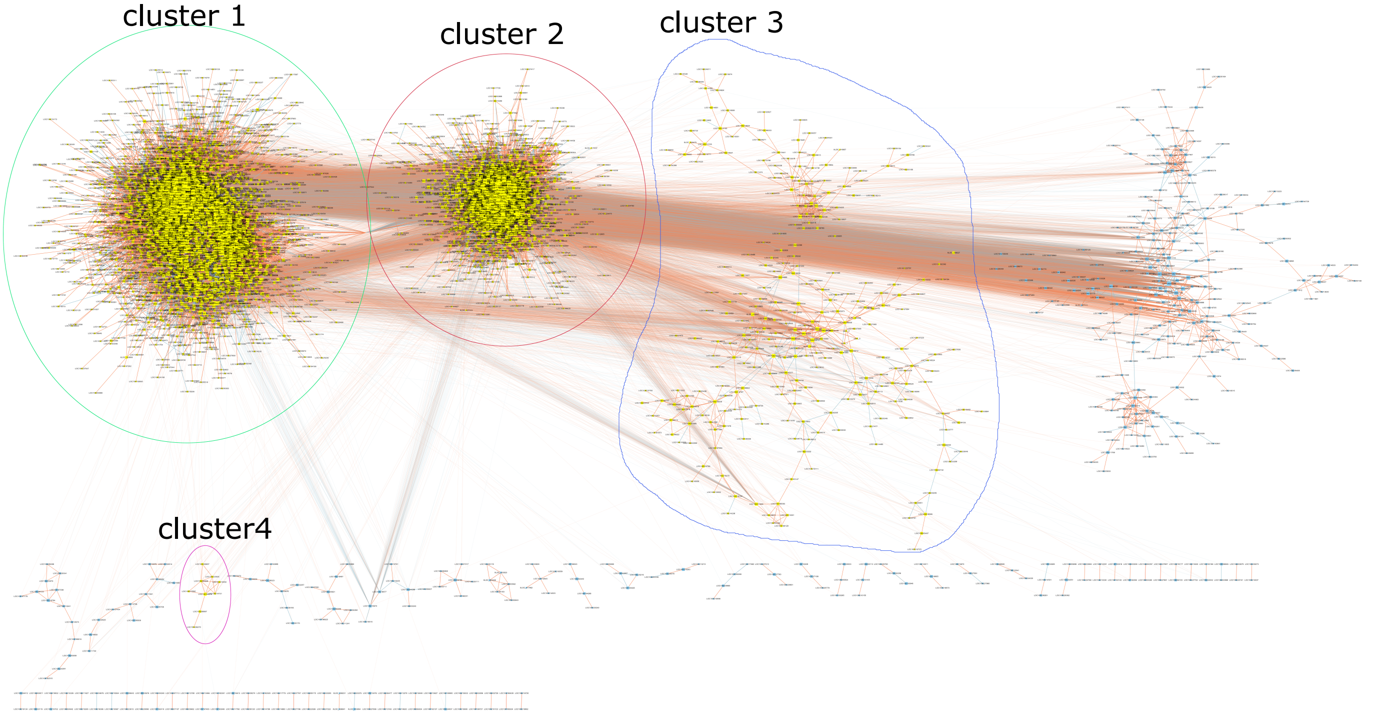

Figure S2. A graph showing a complete co-expression network of genes differentially expressed in carrot storage roots.
